# Supplementary material for: NoxO1 Determines the Level of ROS Formation by the Nox1-Centered NADPH Oxidase
Source: Antioxidants (Basel). 2024 Sep 14;13(9):1113. doi: 10.3390/antiox13091113 (PMC11428687; doi:10.3390/antiox13091113)
Supplement: Supplementary file 1 [file antioxidants-13-01113-s001.zip › antioxidants-3151752-supplementary.pdf]

Supplemental figures

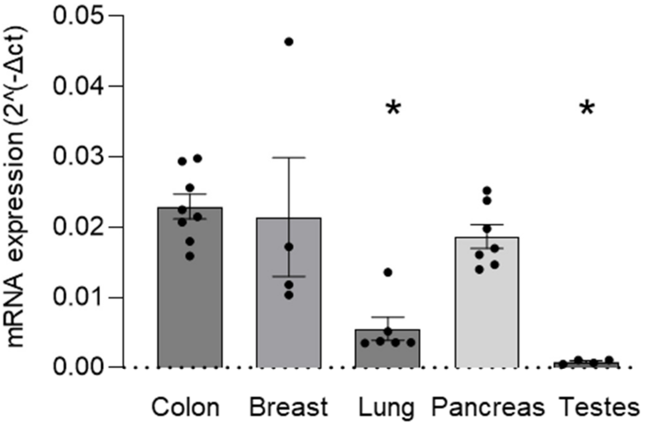

Supplemental figure s1

NoxO1 mRNA expression per copy of GAPDH in murine tissue. 1 biopsy per C75Bl6 mouse (n=4); RT-qPCR in duplicates per sample; mean±SEM; *p<0.05 (NoxO1 mRNA expression in the organ indicated vs. colon)

A

mRNA expression as nTPM and Tau from TCGA human cancer atlas

	CaCo2	MCF7	MDA-MB231	Hek293	Tau
Nox1	29,1	0,4	0,5	1,3	0,85
NoxA1	4,1	18,7	6,6	5	0,52
NoxO1	0,4	0,2	0	0	0,79
Nox2	0	0	0,3	0,3	0,89
p47phox	0,3	0,1	0,2	0,3	0,86
p67phox	0	0	8	0	0,68
p40phox	0	0,3	0	0	0,92
Nox4	0	0	0	0	0,73
Nox5	0,1	0	1,3	0	0,85
Duox1	0,4	0,2	0,3	0,4	0,66
Duox2	2,9	0	0	0	0,84
DuoxA1	0	0	0,3	0	0,81
DuoxA2	1,6	0	0	0	n.d
p22phox	589	958,9	251,7	562,1	0,23
Rac1	220,1	237,1	233,1	194,4	0,1
Rac2	0,4	0,1	211,1	0,6	0,43

B

mRNA expression Hek293

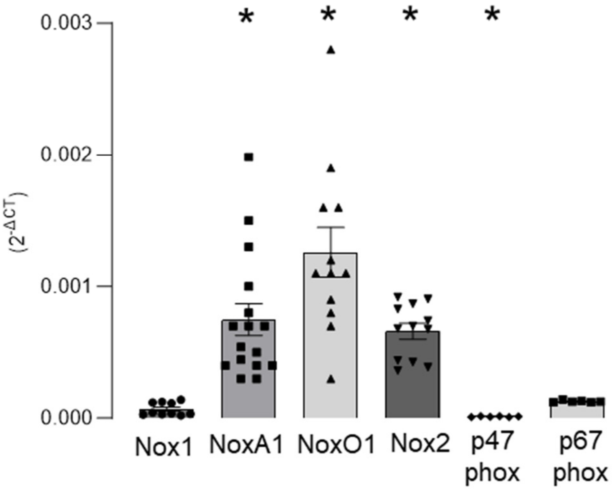

Supplemental figure S2

(A) Table indicating mRNA expression level as nTPM (normalized transcripts per million) as published within the human cancer atlas (TCGA). Tau: Tau specificity score is a numerical indicator of the specificity of the gene expression across cells or tissue with values between 0 (no specificity) to 1 (expression only in one cell type).
(B) mRNA expression of the genes indicated in Hek293 cells. 2^(-ΔCt) to GAPDH (ct=15±1). mean±SEM; n=6; *p<0.05 (mRNA expression of the gene indicated in cells vs. Nox1)

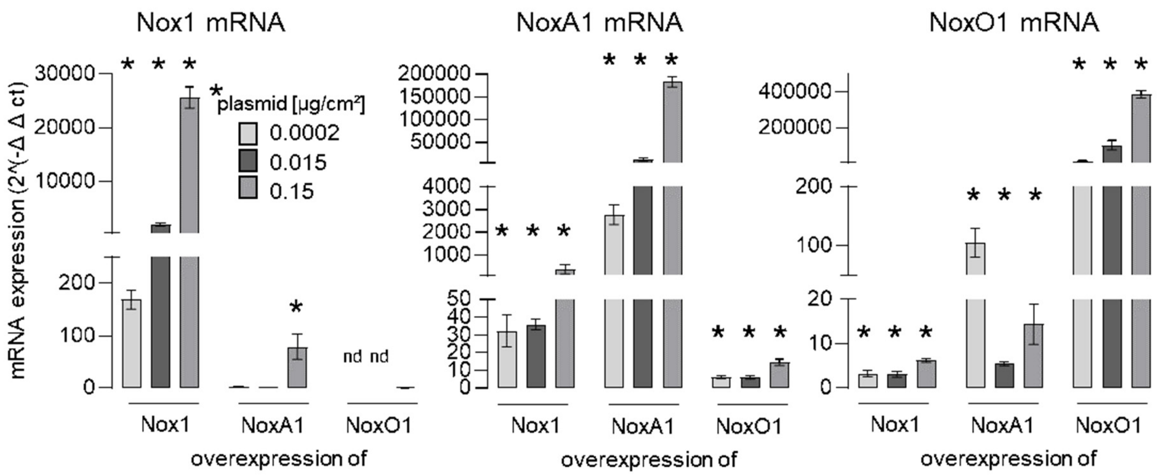

Supplemental figure S3
Nox1, NoxA1 and NoxO1 mRNA expression in Hek293 cells overexpressing single components of the Nox1 centered NADPH oxidase. 2^(-ΔΔCt) to untreated Hek293 cells. mean±SEM; n=6; *p<0.05 (mRNA expression of the gene indicated in cells overexpressing Nox1, NoxA1 or NoxO1 vs. untransfected cells)

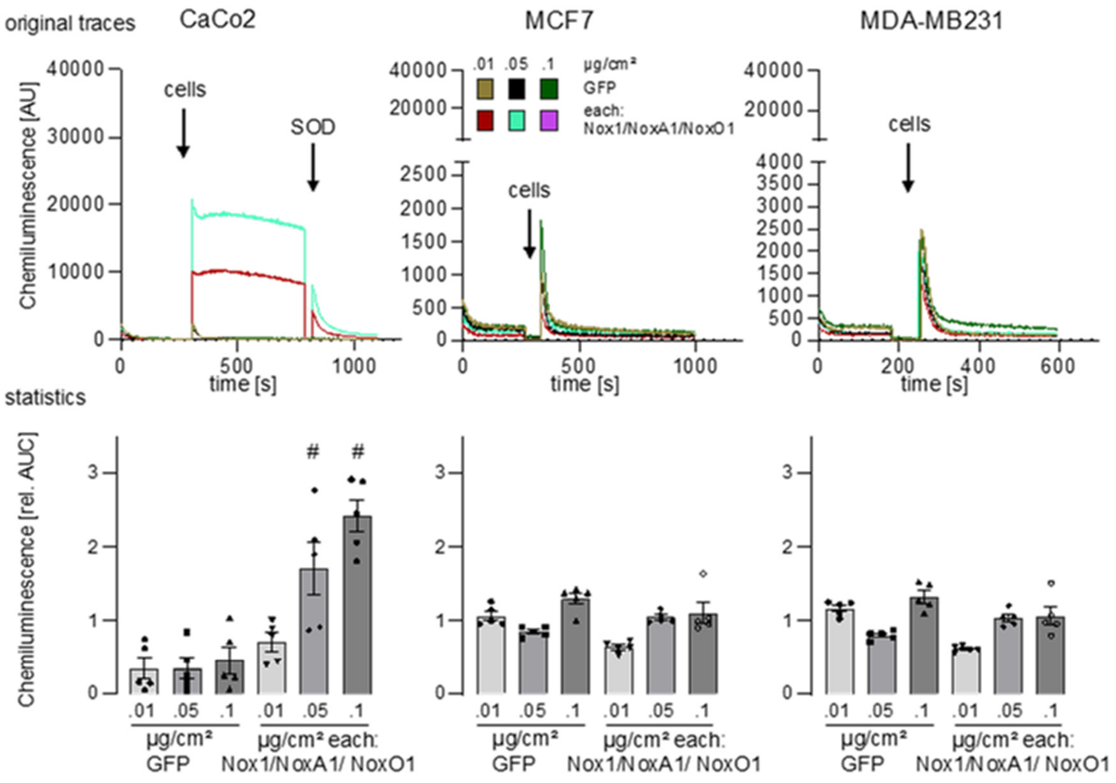

Supplemental figure S4
ROS formation in CaCo2, MCF7 and MDA-MB231 overexpressing increasing plasmid amounts of GFP or Nox1/NoxA1/NoxO1 as indicated. AU - arbitrary units, AUC - area under the curve; ; mean±SEM; n=5; #p<0.05 (ROS formation in cells overexpressing Nox1/NoxA1/NoxO1 vs. GFP transfected cells)

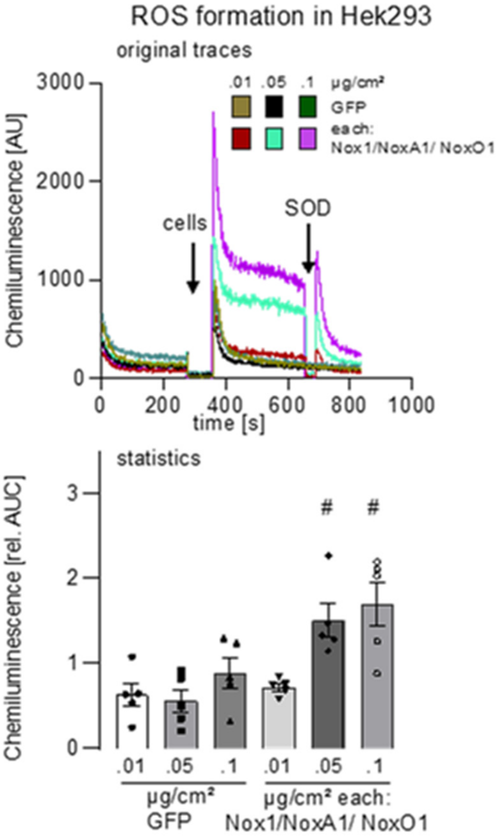

Supplemental figure S5

ROS formation as measured by L-012 mediated chemiluminescence in Hek293 cells over expressing Nox1, NoxA1 and NoxO1 at accelerating concentration as indicated. AU - arbitrary units, rel. AUC - relative area under the curve: 1 equals the mean of all data points per experiment; mean±SEM; n=4; #p<0.05 (ROS formation in cells overexpressing Nox1/NoxA1/NoxO1 or Nox1/NoxA1 vs. GFP transfected cells)

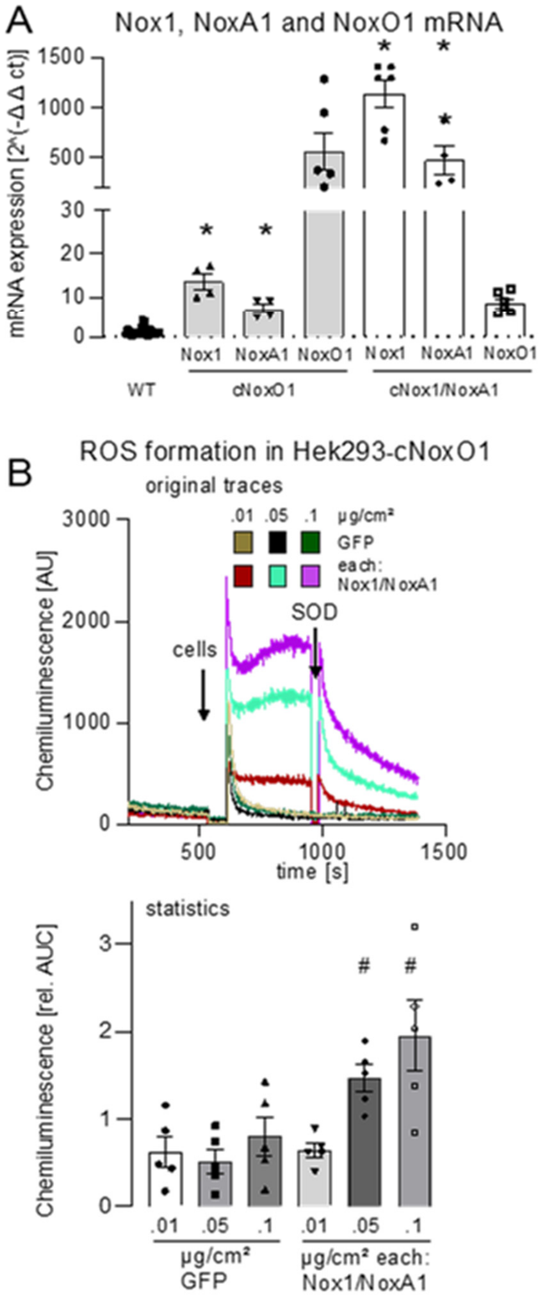

Supplemental figure S6
(A) Nox1, NoxA1 and NoxO1 mRNA expression in Hek293 cells overexpressing single components of the Nox1 centered NADPH oxidase in constitutive NoxO1 overexpressing cells transfected with Nox1/NoxA1. 2^(-ΔΔCt) to untreated Hek293; mean±SEM; n=5; *p<0.05 (mRNA expression of the gene indicated in cells overexpressing Nox1, NoxA1 or NoxO1 vs. untransfected cells) (B) ROS formation as measured by L-012 mediated chemiluminescence in HEK293 with constitutive overexpression of NoxO1 transfected with Nox1 and NoxA1 at accelerating concentration as indicated. AU - arbitrary units, rel. AUC - relative area under the curve: 1 equals the mean of all data points per experiment; mean±SEM; n=4; #p<0.05 (ROS formation in cells overexpressing Nox1/NoxA1 vs. GFP transfected cells)
